# Chimeras of p14ARF and p16: Functional Hybrids with the Ability to Arrest Growth

**DOI:** 10.1371/journal.pone.0088219

**Published:** 2014-02-05

**Authors:** Richard T. Williams, Lisa M. Barnhill, Huan-Hsien Kuo, Wen-Der Lin, Ayse Batova, Alice L. Yu, Mitchell B. Diccianni

**Affiliations:** 1 Department of Pediatric Hematology/Oncology, University of California San Diego, San Diego, California, United States of America; 2 Department of Chemistry and Biochemistry, University of California San Diego, La Jolla, California, United States of America; 3 Genomics Research Center, Academia Sinica, Taipei, Taiwan; Rush University Medical Center, United States of America

## Abstract

The *INK4A* locus codes for two independent tumor suppressors, p14ARF and p16/CDKN2A, and is frequently mutated in many cancers. Here we report a novel deletion/substitution from CC to T in the shared exon 2 of *p14ARF/p16* in a melanoma cell line. This mutation aligns the reading frames of *p14ARF* and *p16* mid-transcript, producing one protein which is half p14ARF and half p16, chimera ARF (chARF), and another which is half p16 and half non-p14ARF/non-p16 amino acids, p16-Alternate Carboxyl Terminal (p16-ACT). In an effort to understand the cellular impact of this novel mutation and others like it, we expressed the two protein products in a tumor cell line and analyzed common p14ARF and p16 pathways, including the p53/p21 and CDK4/cyclin D1 pathways, as well as the influence of the two proteins on growth and the cell cycle. We report that chARF mimicked wild-type p14ARF by inducing the p53/p21 pathway, inhibiting cell growth through G2/M arrest and maintaining a certain percentage of cells in G1 during nocodazole-induced G2 arrest. chARF also demonstrated p16 activity by binding CDK4. However, rather than preventing cyclin D1 from binding CDK4, chARF stabilized this interaction through p21 which bound CDK4. p16-ACT had no p16-related function as it was unable to inhibit cyclin D1/CDK4 complex formation and was unable to arrest the cell cycle, though it did inhibit colony formation. We conclude that these novel chimeric proteins, which are very similar to predicted p16/p14ARF chimeric proteins found in other primary cancers, result in maintained p14ARF-p53-p21 signaling while p16-dependent CDK4 inhibition is lost.

## Introduction

*p16 (CDKN2A)* and *p14ARF* are two overlapping genes in the *INK4A* locus located on chromosome 9p21. They share common exons 2 and 3 yet have different first exons, 1α for *p16* and 1β for *p14ARF*, which are 20 kb apart and under the control of independent promoters. Both code for proteins which regulate the cell cycle and proliferation, albeit through different mechanisms [1]. p16 binds cyclin-dependent kinases 4 and 6 (CDK4/6) and in doing so inhibits the association of cyclin D1 and CDK4/6, thereby preventing phosphorylation of the retinoblastoma protein (Rb) and ultimately slowing cell growth by arresting cells in the G1 phase of the cell cycle. p14ARF, on the other hand, prevents p53 degradation by sequestering the E3 ubiquitin-protein ligase mouse double minute 2 homolog (MDM2). This stabilization of p53 leads to the induction of the cyclin-dependent kinase inhibitor p21 (*CDKN1A*) and subsequently halts cell growth by arresting cells in the G1 and G2/M phases of the cell cycle.

Inactivation of the *INK4A* locus is common across cancers and can be found in up to 95% of pancreatic cancers, 80% of head and neck squamous cell carcinomas and 50% of familial melanomas [2], [3] and [4]. Our group has also reported p16 abrogation rates upwards of 90% in primary T-cell lymphoblastic leukemia, demonstrating the importance of *INK4A* inactivation in the progression of blood cancers as well as solid-tumors [5]. It is for this reason that *CDKN2A* has often been referred to as a susceptibility gene for many cancers [6] and [7]. However, given the tumor suppressing properties of both p16 and p14ARF protein, it is unclear whether inactivation of p16 or p14ARF is more influential on tumor progression. In metastatic melanoma, p16-independent p14ARF inactivation has been found to be frequent, substantiating the role of p14ARF in tumor suppression [8]. On the other hand, studies in familial melanoma have shown a lack of p14ARF inactivation in disease development, hinting that p16 is the main tumor suppressor in the *INK4A* locus [9]. Further complicating the matter, approximately 40% of mutations and deletions at this locus occur in exon 2, affecting both *p16* and *p14ARF* [10]. Such exon 2 mutations can often affect the reading frames of *p14ARF* and *p16* and result in proteins with novel C-termini, though functional data on such proteins is limited and reports are often speculative [11], [12] and [13].

In an effort to elucidate the functional outcome of such *INK4A* frameshift mutations, we describe and functionally characterize a previously unreported mutation in the shared exon 2 of *p16/p14ARF* which we identified in a melanoma cell line. This mutation alters the reading frames of both *p16* and *p14ARF*, creating novel chimeric proteins with specific functionality. We assessed the impact of these proteins on cell cycle regulation and growth suppression to understand their potential roles in cancer progression.

## Methods and Materials

### Cell Culture and Reagents

The melanoma cell line M2 was a gift from Dr. Malcolm Mitchell and Dr. Boris Minev at the University of California, San Diego and its origin has been previously published [14]. U2OS cells were obtained from the American Type Culture Collection (ATCC HTB-96). Cells were cultured in RPMI 1640 with 10% (v/v) fetal bovine serum, 100 U/ml penicillin, 100 µg/ml streptomycin, and 2 mM l-glutamine in a humidified incubator at 37°C with 5% CO_2_. Nocodazole was purchased from Sigma.

### DNA Sequencing and Cloning

For transcript sequencing, 2 µg of RNA was reverse transcribed into cDNA using the Invitrogen Superscript III First Strand Synthesis System. *p14ARF* was amplified using primers ARF-bc-35F (5′-CTCAGGGAAGGCGGGTGCGC-3′) and 3p16T (5′-CTACGAAAGCGGGGTGGGTTGT-3′). *p16* was amplified using primers E1S (5′-TGGCTGGTCACCAGAGGGTGGGG-3′) and 3p16T. PCR reactions took place in a 50 µl volume with 1 µl of cDNA, 10 pmol each of sense and antisense primer, 200 µM dNTPs, 1.5% DMSO, and 1 U Phusion High Fidelity DNA Polymerase (New England BioLabs) in 1x Phusion HF Buffer. Amplified products were purified over Qiagen QiaQuick PCR Purification Columns and sequenced at the Moores UCSD Cancer Center DNA Sequencing Shared Resource using the reverse sequencing primer c531R (5′-CTAAGTTTCCCGAGGTTTCT-3′).The *p14ARF* and *p16* transcripts from M2 were subcloned into the pcDNA3.1 Expression Vector (Invitrogen) for artificial expression.

### Transfection

Transient transfection of expression constructs into U2OS cells was performed using the Neon Transfection System (Life Technologies) according to the manufacturer's protocol. Cells were washed in PBS and detached using trypsin. 2×10^6^ cells were washed twice with PBS and centrifuged 10 minutes at 300xg. Cells were resuspended in 100 µl Buffer R, mixed with 10 µg plasmid DNA, and electroporated with 4 pulses at 1230 volts and a pulse length of 10 ms. Cells were then moved to T-25 flasks containing RPMI 1640 media with 10% fetal bovine serum and no antibiotics. Cells were allowed to adhere for 48 hours before fixing for cell cycle analysis or harvesting for protein. The transfection efficiency of U2OS cells was determined to be 85–90% by transfecting cells with a GFP expression construct and counting fluorescent cells 48 hours later under fluorescent microscopy.

### Immunoblotting

Cells were lysed in RIPA buffer (50 mM Tris-HCl pH 8.0, 1% Triton X-100, 150 mM NaCl, 1 mM EDTA, 0.5% Deoxycholate, 0.1% Sodium Dodecyl Sulfate, 1 mM Sodium Fluoride, 1 mM Sodium Pyrophosphate, 1 mM PMSF and 1x Protease Inhibitor Cocktail from Sigma). Lysates were clarified by centrifugation at 10,000xg for 15 minutes at 4°C and mixed with 4x NuPAGE LDS sample buffer (Life Technologies) and 0.05 M Dithiothreitol, heated 10 minutes at 80°C, then separated by SDS-PAGE on a 10% Bis-Tris NuPAGE Gel (Life Technologies) and transferred to PVDF (Bio-Rad). PVDF membranes were blocked with 5% Bovine Serum Albumin (Sigma) in TBS with 0.05% Tween 20 and probed with antibodies against p16 C-terminus (C-20; 1∶400; Santa Cruz Biotechnology), p16 N-terminus (N-20; 1∶100; Santa Cruz Biotechnology), p14ARF N-terminus (NB200-111; 1∶400; Novus Biologicals), CDK4 (H-22; 1∶400; Santa Cruz Biotechnology), cyclin D1 (CD1.1; 1∶200; Santa Cruz Biotechnology), p53 (PAb1801; 1∶800; Thermo Scientific), p21 (6B6; 1∶400; BD Pharmingen), and β-actin (AC-15; 1∶100,000; Sigma). An HRP-conjugated mouse antibody (1∶1000; Kirkegaard & Perry Laboratories) or HRP-conjugate Protein A (1∶3000; BioRad) was used as a secondary detection probe. Bands were visualized using ECL enhanced chemiluminescent substrate (Pierce) and exposed to HyBlot CL Autoradiography film (Denville Scientific). Film was developed with a Kodak film developer.

### Co-Immunoprecipitation

Cells were lysed in a non-denaturing lysis buffer (50 mM Tris-HCl pH 8.0, 150 mM NaCl, 10% Glycerol, 1% Igepal CA-630, 1 mM EDTA, 1 mM Sodium Fluoride, 1 mM PMSF and 1x Protease Inhibitor Cocktail from Sigma). The lysate was clarified by centrifugation at 10,000xg for 15 minutes at 4°C. 500 µg of protein was incubated with 2 µg of anti-CDK4 antibody (H22) overnight at 4°C with rocking. After the overnight incubation, 50 µl of Protein G Dynabeads (Life Technologies) suspended 1∶1 in non-denaturing lysis buffer was added to the lysate and incubated 4 more hours at 4°C with rocking. The Dynabeads were washed four times with ice cold lysis buffer, then resuspended in 20 µl of 1x LDS sample buffer (Life Technologies) with 0.05 M dithiothreitol and incubated 10 minutes at 80°C to release bound protein. Released protein was analyzed side by side with 8.5 µg of lysate by immunoblotting as described above.

### Cell Cycle Analysis

After transfection, cells were allowed to adhere for 24 hours before treating with or without 100 ng/ml nocodazole for an additional 24 hours. Cells were fixed in 70% ethanol overnight at −20°C and then stained for 15 minutes at 37°C with propidium iodide (PI) staining solution (30 µg/ml propidium iodide, 200 µg/ml DNase-free RNase A, and 0.1% Triton X-100). PI content of cells was measured using a BD FACSCalibur Flow Cytometer and cell cycle distribution was determined using FlowJo Analysis Software.

### Cell Proliferation Assays

Cell growth was determined 48 hours after plating cells in T-25 flasks. Cells were washed with PBS, detached using trypsin, mixed with trypan blue to assess viability and counted. Colony formation assays were performed as previously described [15]. Briefly, U2OS cells were transfected with each expression plasmid or a negative control plasmid not containing the G418 resistance gene. After 48 hours cells were trypsinized, washed in PBS, and counted. 3000 cells were seeded in 60-mm culture dishes in triplicate. After 24 hours, plated cells were treated with 400 µg/ml of G418 (Life Technologies) for 4 weeks and allowed to form colonies. Colonies were fixed with 0.3% glutaraldehyde and stained with 1% methylene blue. Colonies larger than 1 mm in diameter in each dish were counted and defined as colony forming units (CFU). CFU values were normalized to percent of pcDNA3.1 vector control.

## Results

### CC to T mutation in exon 2 of *p16/p14ARF* yields two chimeric proteins

**Figure 1:**
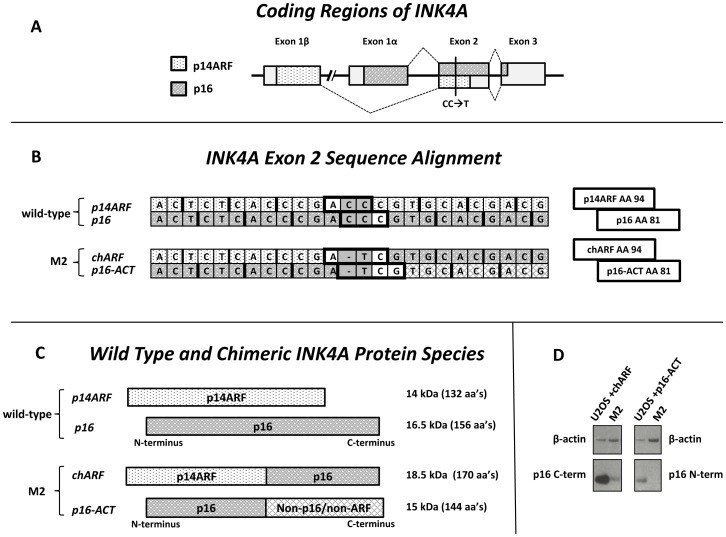
chARF and p16-ACT arise from a CC to T mutation in exon 2 of *INK4A*. (A) *p16* and *p14ARF* are initiated from exons 1α and 1β, respectively, but share exons 2 and 3. The CC to T mutation found in the M2 cell line is shown by a black bar. Patterned areas represent respective coding regions. (B) Comparison of wild-type *p14ARF* and *p16* transcripts versus the *chARF* and *p16-ACT* transcripts derived from M2 cells. Reading frames are noted by single bold lines. Codons 94 and 81 are boxed in bold for p14ARF and p16, respectively. The reading frame of chARF is switched from the p14ARF reading frame to the p16 reading frame after codon 94 of p14ARF, codon 81 of p16. At the same respective codon, the reading frame of p16-ACT changes from a p16 reading frame to a non-p16/non-p14ARF reading frame. (C) The proteins resulting from wild-type and M2 *INK4A* transcripts are shown for comparison. chARF contains the amino terminus (N-terminus) of p14ARF and the carboxyl terminus (C-terminus) of p16. p16-ACT contains the amino terminus of p16 with a non-p16/non-p14ARF carboxyl terminus. (D) Western blot of chARF and p16-ACT in M2 cells. U2OS cells were transfected with chARF or p16-ACT and used as controls. β-actin is shown as a control for loading differences. chARF protein was detectable in M2 cells while p16-ACT was undetectable.

While characterizing the gene status of *p16* and *p14ARF* in the melanoma cell line M2, we sequenced *p16* and *p14ARF* transcripts in M2 cDNA and found a CC to T mutation at codon 81 of *p16* (codon 94 of *p14ARF*) (**Fig. 1A, B**). No wild-type transcripts were detected and sequencing of M2 genomic DNA likewise revealed only the mutated sequence with no evidence of a wild-type allele. This deletion/substitution caused the *p14ARF* transcript to switch to the *p16* reading frame after codon 81 of *p16*, while the *p16* transcript switched to a non-*p16*/non-*p14ARF* reading frame (**Fig. 1B**). The transcript driven off the *p14ARF* promoter coded for the first 93 amino acids of p14ARF, one missense amino acid, and 75 amino acids of p16 (chimera ARF [chARF]). The transcript driven off the *p16* promoter coded for the first 80 amino acids of p16 followed by 64 non-p16/non-p14ARF amino acids (p16-Alternative Carboxyl Terminus [p16-ACT]) (**Fig. 1C**). Nucleotide sequences for the two transcripts were deposited in the NCBI GenBank nucleotide database (accession no. KC806059 and KC814157 for *chARF* and *p16-ACT*, respectively). M2 cell lysate was then analyzed via Western blot to determine if *chARF* and *p16-ACT* transcripts form stable proteins in M2 cells. chARF protein was successfully detected in M2 lysate using a C-terminal directed p16 antibody while p16-ACT was unable to be detected using an N-terminal directed p16 antibody (**Fig. 1D**).

### chARF increases levels of p53 and p21 protein

**Figure 2:**
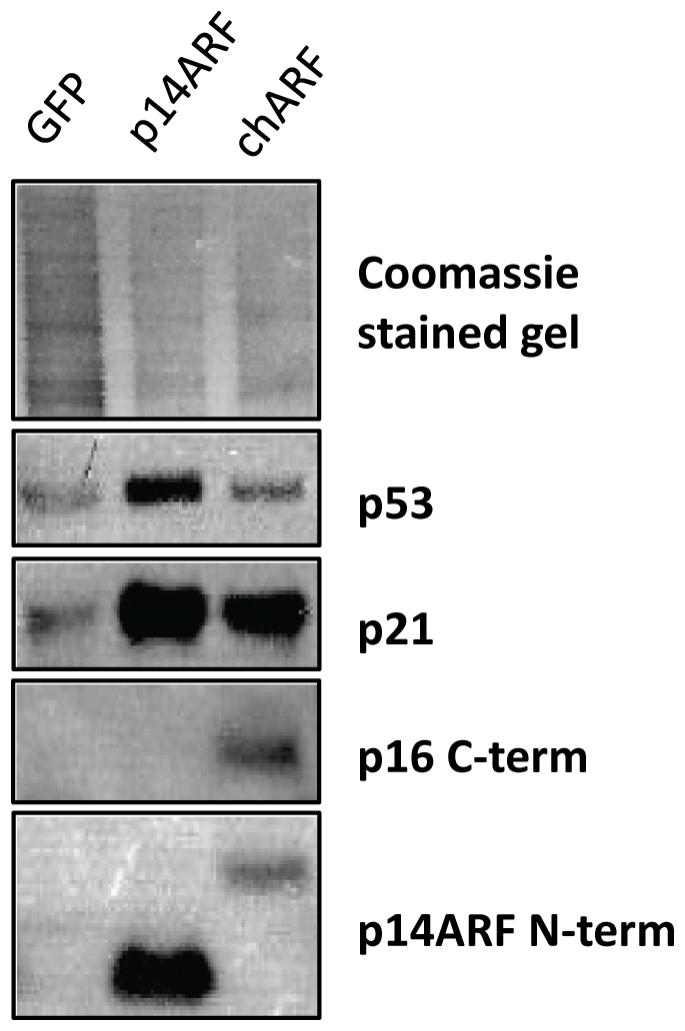
chARF activates the p53/p21 pathway. U2OS osteosarcoma cells were transfected with chARF or p14ARF and analyzed for p53 and p21 protein expression by Western Blot. p14ARF and chARF expression were verified with antibodies to the amino terminus (N-term) of p14ARF (detects p14ARF and chARF) or the carboxyl terminus (C-term) of p16 (detects chARF only). As a loading control, total protein in the SDS-PAGE gel was stained with coomassie brilliant blue. P53 and p21 protein were elevated in both chARF and p14ARF transfected cells as compared to GFP control.

As chARF shares 50% of its amino acid sequence with wild-type p14ARF, we asked whether chARF held p14ARF functionality in the p53/p21 pathway. The U2OS osteosarcoma cell line was chosen as a model system for studying the effects of chARF because these cells are wild-type for both *p53* and *p21* but are *p14ARF/p16* inactivated. Further, U2OS cells are sensitive to growth inhibition by transfected wild-type *p16* and *p14ARF* [15] and [16]. U2OS cells were transfected with expression constructs containing *chARF*, *p14ARF*, or *GFP* (control) cDNA and after 24 hours total protein was analyzed via Western blot. chARF and p14ARF protein expression were verified using antibodies against the C-terminus of p16 or the N-terminus of p14ARF. Total protein on the SDS-PAGE gel was stained with Commassie brilliant blue and visualized to account for loading differences. As predicted by sequence analysis, chARF protein had an apparent molecular weight of 19 kDa, slightly larger than the 14 kDa p14ARF. Results showed that expression of both chARF and p14ARF protein lead to marked increases in p53 and p21 protein when compared to the GFP transfected control, demonstrating that chARF can function as wild-type p14ARF in the p53/p21 pathway (**Fig. 2**).

### chARF binds CDK4, but does not prevent cyclin D1 binding

**Figure 3:**
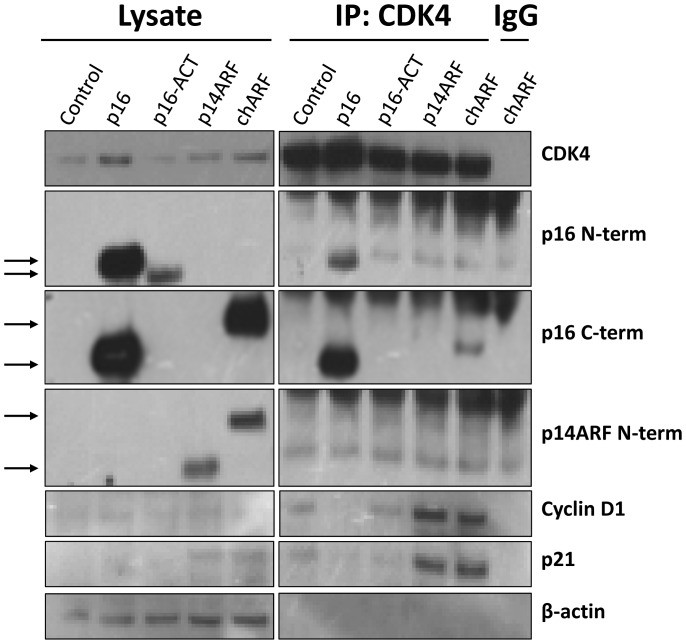
Neither chARF nor p16-ACT prevents Cyclin D1 from binding CDK4. U2OS cells were mock transfected (control) or transfected with p16, p16-ACT, p14ARF or chARF expression constructs and CDK4 was immunoprecipitated under non-denaturing conditions. Immunoprecipitations were analyzed via immunoblot for the presence of CDK4, Cyclin D1, p16, p16-ACT, p14ARF, chARF and p21. The PVDF membrane was cut horizontally at the 25 kDa molecular weight marker and the top half was probed with antibodies against CDK4, Cyclin D1, and β-actin while the bottom half was probed with antibodies for p16, p14ARF, and p21. chARF (19kDa) was detected with antibodies to the amino-terminus (N-term) of p14ARF or the carboxyl terminus (C-term) of p16. p16-ACT (15 kDa) was detected with an antibody to the amino terminus (N-term) of p16. β-actin was measured in the lysate as a loading control. Immunoprecipitations are shown on the right half of the blot (IP:CDK4) while input lysates are shown on the left (Lysate). Immunoprecipitation using normal rabbit IgG was performed on chARF transfected U2OS cells (IgG, far right) to ensure target proteins were not immunoprecipitated nonspecifically. A comparable amount of CDK4 was immunoprecipitated in each CDK4 IP, indicating that differing amounts of co-immunoprecipitated Cyclin D1 was a result of the transfected proteins. chARF was detected in the CDK4 IP using a C-term p16 antibody while p16-ACT was not detected in the CDK4 IP using the N-term p16 antibody. Only wild-type p16 prevented Cyclin D1 from binding CDK4 compared to control. In p14ARF and chARF transfected cells, induction of p21 stabilized the Cyclin D1/CDK4 complex, resulting in greater amounts of co-immunoprecipitated Cyclin D1 compared to control.

p16-ACT contains Ankyrin repeats I and II of full length p16 while chARF has most of repeat III and all of repeat IV. Based on biochemical studies which demonstrate that repeats I and II do not fold correctly in the absence of repeats III and IV [17] and [18], we hypothesized that the p16 portion of p16-ACT would be insufficient to bind CDK4 while the p16 portion of chARF would correctly bind CDK4 and prevent the binding of cyclin D1. To test this, p16, p16-ACT, p14ARF, chARF, or GFP (Control) proteins were expressed in U2OS cells and CDK4 was immunoprecipitated under non-denaturing co-immunoprecipitation (co-IP) conditions. The immunoprecipitation (IP) was separated by SDS-PAGE before immunoblotting for CDK4, cyclin D1, p14ARF/p16 species or β-actin. IP reactions are shown in the right portion of the blot (IP:CDK4) while the input lysates are shown on the left (Lysate) (**Fig. 3**). The IP was also carried out using a rabbit IgG control antibody (IgG; far right on blot) in chARF transfected cells to ensure that no proteins were immunoprecipitated nonspecifically. Expression of p16, p16-ACT, p14ARF or chARF protein was verified by probing the lysate with antibodies specific to the N-terminus of p16 (detecting wild-type p16 and p16-ACT), the C-terminus of p16 (detecting wild-type p16 and chARF), or the N-terminus of ARF (detecting p14ARF and chARF). The ability of the transfected proteins to bind CDK4 was determined by probing the IP lanes with the same antibodies. As expected, p16-ACT ran at a slightly smaller molecular weight than p16, with a predicted and apparent molecular weight of 15 kDa. As seen in the IP lanes, p16 co-immunoprecipitated with CDK4 very efficiently (**Fig. 3**). chARF was detected in the CDK4 IP using a p16 C-terminal directed antibody, though to a lesser extent than wild-type p16. chARF was unable to be detected in the IP using an antibody against p14ARF, likely due to inferior sensitivity of the p14ARF antibody. p16-ACT was not found in the IP, though it is unclear whether this was because p16-ACT protein expression was too low for detection or if the amino-terminal p16 portion was insufficient to bind CDK4 (**Fig. 3**).

Comparable amounts of CDK4 were immunoprecipitated across all samples, ensuring that any changes in cyclin D1 binding were not due to loading differences. Compared to the GFP control, p16 inhibited the formation of a cyclin D1/CDK4 complex as expected. (**Fig. 3**). Meanwhile, the cyclin D1 level in the p16-ACT transfected IP sample was comparable to the control, further indicating that p16-ACT does not function to inhibit the formation of the CDK4/cyclin D1 complex. Again, it is uncertain whether this is because of its low protein expression compared to wild-type p16 or because it does not contain the necessary amino acid sequence. chARF, though able to bind CDK4, also did not prevent cyclin D1 from binding to CDK4. On the contrary, cyclin D1 levels in the CDK4 IP for both chARF and p14ARF transfected cells were markedly higher than that of the control. This seemed counterintuitive to the negative growth effects seen in p14ARF-transfected cells. However, while p21 inhibits CDK2/cyclin E complexes to arrest cells at the G1/S transition, it has also been shown to play a role in the positive regulation of CDK4/cyclin D complexes in G1 [19] and [20]. Considering that chARF mimicked p14ARF in inducing p53 accumulation and subsequent p21 induction, we hypothesized p21 was binding and stabilizing the CDK4/cyclin D1 complex in both p14ARF and chARF transfected cells. Testing this idea, we probed for p21 in the CDK4 co-IP and found significant amounts of p21 in complex with CDK4 in chARF and p14ARF transfected samples, mirroring the pattern of increased cyclin D1 binding (**Fig. 3**).

### chARF causes potent growth arrest of U2OS cells while p16-ACT has little effect

**Figure 4:**
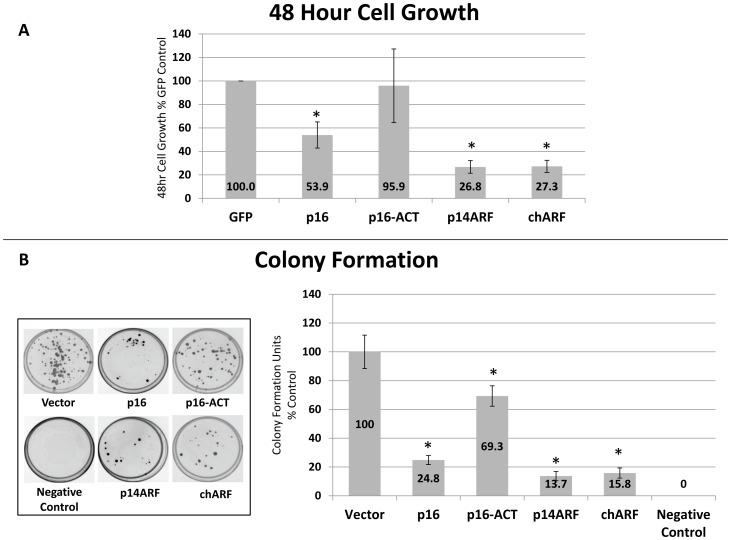
chARF slows the growth of U2OS cells. (A) U2OS cells were transfected with p16, p16-ACT, p14ARF, chARF, or GFP control. After 48 hours, cells were counted using Trypan blue to assess cell number and viability. p14ARF and chARF reduced cell growth by 73% compared to GFP control cells (p = 0.002 two-tailed unpaired T-test compared to GFP control) while p16 reduced growth by 46% (p = 0.02 two-tailed unpaired T-test compared to GFP control). p16-ACT had no significant effect on growth (p = 0.8 two-tailed unpaired T-test compared to GFP control). (B) Cells were transfected with p16, p16-ACT, p14ARF, chARF, empty vector (Vector), or a negative control vector without the G418 selection gene (Negative Control) and grown in 400 µg/ml G418 for four weeks. Colonies were the stained and counted. Percent colony formation is expressed as a percent of the vector control. p14ARF and chARF reduced colony formation by 86% and 84%, respectively, compared to vector control (p = 0.006 two-tailed unpaired T-test). p16 was slightly less potent, slowing colony formation by 75% (p = 0.008 two-tailed unpaired T-test compared to vector control) while p16-ACT slowed colony formation by 31% (p = 0.04 two-tailed unpaired T-test compared to vector control). Right: the average of three replicate experiments with standard deviation. *p<0.05 two-tailed unpaired T-test. Left: plates of a representative experiment showing stained colonies.

After defining their molecular function, we sought to characterize the ability of chARF and p16-ACT to regulate growth. U2OS cells were transfected with p16, p16-ACT, p14ARF, chARF or GFP control constructs and after 48 hours cells were counted in Trypan blue to determine viable cell number. Percent growth over 48 hours was normalized to GFP control. p16 transfected cells had a 46% reduction in growth as compared to GFP (p = 0.02 two-tailed unpaired T-test) (**Fig. 4A**). chARF and p14ARF both potently affected growth, showing a 73% reduction in growth in each case (p = 0.002 two-tailed unpaired T-test). p16-ACT had no significant effect on growth when compared to GFP control (p = 0.8 two-tailed unpaired T-test).

Given that the growth experiments described above can only be carried out over short incubation times, it was uncertain if more time was needed for p16-ACT to exert subtle effects on growth. We therefore also measured growth by colony formation assay, which allows cells to grow for four weeks. U2OS cells were transfected with constructs for p16, p16-ACT, p14ARF, chARF, empty pcDNA3.1 vector with G418 resistance (Vector), or Phr-GFP-c vector without G418 resistance (Negative control) and colony formation was evaluated after four weeks. Colony formation units (CFU) were translated into percentages of vector control and graphed accordingly (**Fig. 4B**). Colony plates are also shown from a representative experiment. Cells containing the Phr-GFP-c vector were unable to grow in the selective media, proving the effectiveness of the selection agent. As in our other growth experiments, chARF and p14ARF had potent and nearly identical growth effects, reducing colony formation by 84 and 86%, respectively, compared to vector control (p = 0.006 two-tailed unpaired T-test). p16 also had a more pronounced effect on growth in this assay, showing a 75% reduction in colony formation (p = 0.008 two-tailed unpaired T-test). Surprisingly and in seeming opposition to the lack of growth effects seen after 48 hours, p16-ACT reduced colony formation by 31% when compared to the vector control (p = 0.04 two-tailed unpaired T-test) (**Fig. 4B**).

### chARF mimics p14ARF in arresting cells in G1 and G2/M

**Figure 5:**
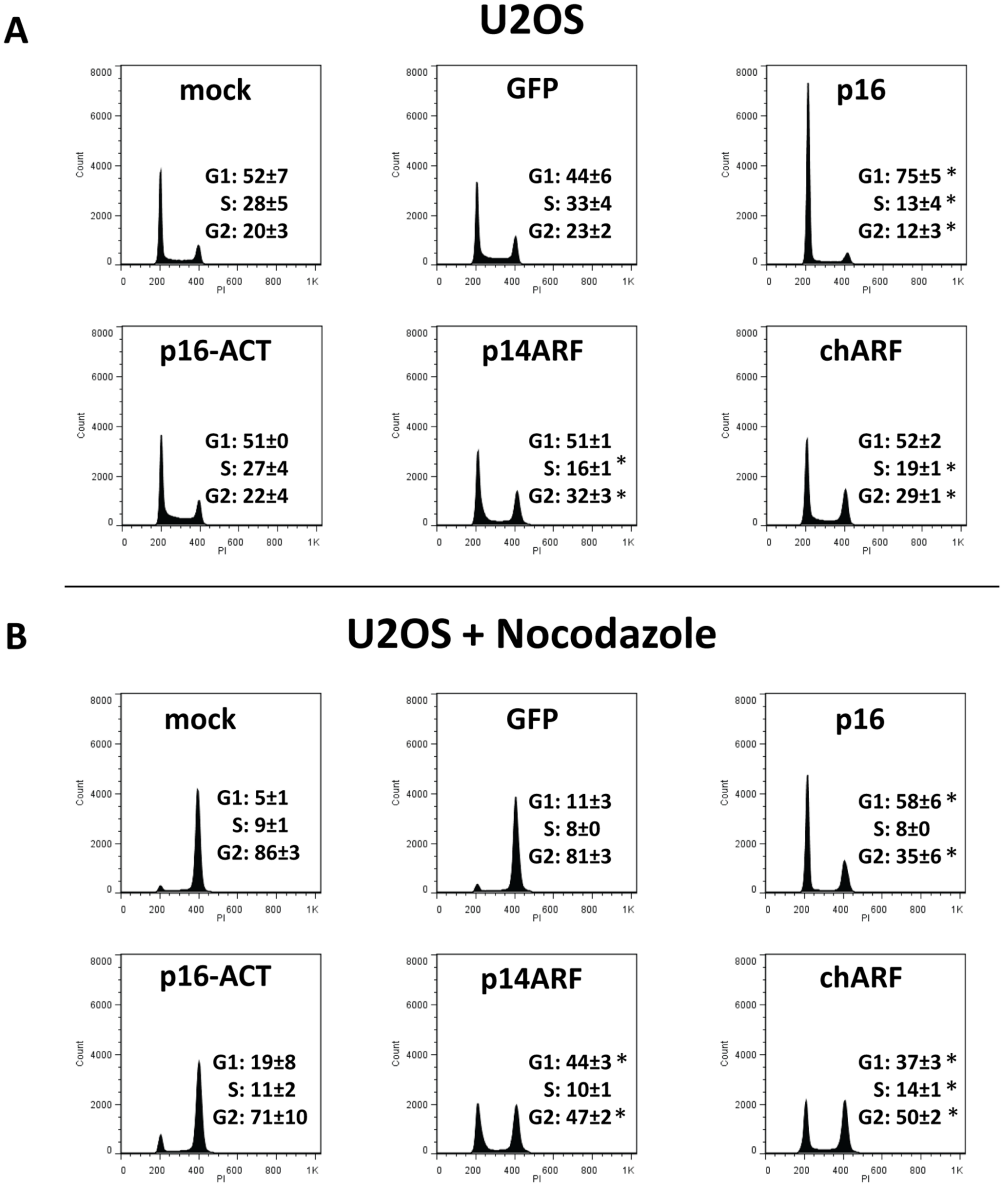
chARF arrests U2OS cells in the G1 and G2/M phases of the cell cycle. U2OS cells were transfected with p16, p16-ACT, p14ARF, chARF, GFP, or no DNA (mock). 24 hours after transfection, cells were (A) left untreated or (B) treated with 100 ng/ml nocodazole to induce G2/M arrest, highlighting any G1 maintenance while distinguishing between G1 arrest versus shortening of the S and G2/M phases. 48 hours after transfection, cells were fixed, stained for total DNA content using propidium iodide and analyzed by flow cytometry. Cell cycle distribution was determined using FlowJo analysis software. Histograms show the G1 and G2/M peaks (2 n diploid and 4 n quadraploid cells, respectively) along with percent of relative distribution frequencies. p16 transfected cells showed a stark G1 arrest when untreated and were resistant to G2/M arrest by nocodazole. p14ARF and chARF transfected cells had identical cell cycle distributions, showing G2/M arrest when untreated and maintenance of G1 levels when nocodazole treated. The cell cycle distribution of p16-ACT transfected cells was indistinguishable from that of the controls under both untreated and nocodazole treated conditions. Values shown are the average of two replicate experiments with standard deviation. *p<0.05 Wald's test. Histograms shown are from a representative experiment.

p16 and p14ARF both arrest growth by arresting the cell cycle. p16 arrests cells in G1 while p14ARF is reported to arrest cells in G1 and G2/M [21] and [22]. To determine whether chARF and p16-ACT can also induce cell cycle arrest, U2OS cells were transfected with p16, p16-ACT, p14ARF, chARF, GFP, or no DNA (mock) and after 48 hours cells were fixed and stained for total DNA content using propidium iodide. Cells were then analyzed by flow cytometry to determine DNA content and cell cycle distribution (**Fig. 5**). Both GFP and mock controls were included since introducing DNA during transfection can itself affect cell cycle distribution, though in these experiments the two controls were not significantly different (G1 [p = 0.95], S [p = 0.84] and G2/M [p = 0.97]; Wald's test) and therefor GFP and mock control values were combined for statistical analysis. As expected, p16 transfected cells showed G1-arrest compared to controls (p = 0.009; Wald's test compared to controls) (**Fig. 5A**). p16-ACT transfected cells appeared identical to controls in their cell cycle distribution, demonstrating the lack of effect p16-ACT has on the cell cycle. Meanwhile, chARF and p14ARF behaved identically, arresting cells in G2/M (p = 0.02 for chARF and p = 0.007 for p14ARF; Wald's test compared to controls) while decreasing the percentage of cells in S phase (p = 0.03 for chARF and p = 0.02 for p14ARF) but not significantly effecting G1 distribution compared to control.

Having expected that p14ARF would induce G1 as well as G2/M arrest, but also knowing from our CDK4 IP experiments that p14ARF-induced p21 may be enhancing CDK4 activity, we were curious if p14ARF was functioning to maintain a certain percentage of cells in G1 rather than inducing indiscriminate arrest. The experiment was repeated, this time adding 100 ng/ml of the microtubule depolymerizing agent nocodazole to the cells 24 hours after transfection to arrests cells in G2/M. If p14ARF stabilizes G1 in U2OS cells, there should be some resistance to G2/M arrest. Inducing transfected cells to accumulate in G2/M also helps to distinguish between G1 arrest and a shortening of the S and G2/M phases [23]. Nocodazole treatment arrested 80% of both GFP transfected and mock transfected control cells in G2/M, depleting the G1 population to less than 10% of the total (**Fig. 5B**). As expected, p16 transfected cells were very resistant to G2/M arrest by nocodazole, showing significant G1 arrest compared to nocodazole treated controls (p = 0.0002; Wald's test). Notably, chARF and p14ARF transfected cells were also resistant to nocodazole-induced G2 arrest and their G1 phase distributions after nocodazole treatment were markedly greater than controls (p = 0.0007 for chARF and p = 0.0003 for p14ARF; Wald's test), demonstrating G1 regulation that went unseen in experiments without nocodazole. The cell cycle distribution of chARF transfected cells was again nearly identical to that of p14ARF transfected cells, further demonstrating that chARF functions identically to p14ARF. p16-ACT transfected cells showed strong G2/M arrest indistinguishable from that of nocodazole treated controls, giving more evidence that p16-ACT had no influence on cell cycle distribution.

## Discussion

Mutations predicted to create p16-14ARF chimeric proteins have been documented in many primary cancers [11] and [24] including familial melanoma [25], squamous cell carcinoma of the skin [26] and squamous cell carcinoma of the esophagus [13] and therefore such mutations represent an important avenue of cancer research. However, due to the paucity of functional data available, the significance of such mutations has remained mostly speculative. In this study, we report a unique 1 bp deletion, 1 bp substitution in exon 2 of *p16/p14ARF* found in a melanoma cell line which creates two unique p16/p14ARF chimeras. By realigning the reading frames of *p16* and *p14ARF*, the mutation resulted in one protein containing the amino terminus of p14ARF along with the carboxyl terminus of p16, chARF, and another protein containing the amino terminus of p16 with 64 non-p16/non-p14ARF amino acids at its carboxyl terminus, p16-ACT. We studied the effects of these proteins on the cell cycle and cell growth and dissected the molecular mechanisms which bring about these effects in order to determine the functional impact of such frameshift-inducing *INK4A* mutations.

Using *in vitro* functional studies, we showed that chARF mimicked p14ARF by inducing p53 and p21 accumulation, slowing cell growth and regulating the transition out of G1 and G2/M. Unexpectedly, chARF and p14ARF did not induce significant G1 arrest like p16. Rather, chARF and p14ARF demonstrated G1 maintenance during nocodazole-induced G2/M arrest. While others have reported that p14ARF induces G1 arrest [22], our studies give evidence that p14ARF and chARF serve to regulate the progression out of G1 rather than to indiscriminately arrest cells in this phase of the cell cycle. This notion is consistent with the role of downstream p21 as both a CDK4 activator and a CDK2 inhibitor. G1 regulation by p14ARF/p21 therefore appears to be much more fine-tuned than the broad arrest brought on by p16. The fact that p14ARF and chARF slowed U2OS cell growth without inducing G1 arrest also highlights that in contrast with p16 which halts growth by means of G1 arrest, chARF and p14ARF slowed growth mainly by G2/M arrest.

In immunoprecipitation studies, chARF functioned as p16 by binding CDK4. However, chARF did not prevent cyclin D1 from binding CDK4. Rather, the CDK4/cyclin D1 complex was stabilized by chARF-induced p21, overcoming any cyclin D1-displacing potential of chARF. While prior studies have demonstrated that the first 20 amino acids of p14ARF are sufficient to bind MDM2 and prevent p53 degradation [27], here we demonstrated that the p16 portion of our p16-p14ARF chimera did not significantly inhibit or enhance its p14ARF-related activity. This evidence, along with the CDK4 immunoprecipitation experiments showing that chARF was also able to bind CDK4, indicates that both the p16 portion and the p53-affecting p14ARF portion of chARF were able to fold correctly and independently of each other. Because chARF also induced downstream p21 which bound and stabilized the CDK4/cyclin D1 complex, it is unclear whether chARF completely lacked the ability to inhibit cyclin D1 from binding CDK4 or whether this ability was masked by p21-dependent complex stabilization. In support of chARF being unable to inhibit CDK4/cyclin D1 complex formation, p16 has been shown to displace p21 from the CDK4/cyclin D1 complex, freeing up the pool of p21 protein to then bind and inhibit CDK2/cyclin E complexes and further inhibit DNA synthesis [28]. If chARF was capable of binding CDK4 like full length p16, chARF should have displaced p21 from CDK4. Still, as chARF did not bind CDK4 as efficiently as full length p16, it cannot be ruled out that chARF had a slight capacity to displace p21 and prevent cyclin D1 binding but this was overcome by extremely high levels of p21 induced by chARF overexpression. In any case, the overall effect of chARF was enhanced CDK4/cyclin D1 complex stability. This evidence, along with our data in which chARF did not induce G1 arrest like p16, suggests chARF cannot function as wild-type p16.

p16-ACT was unable to prevent cyclin D1/CDK4 complex formation, unable to affect cell cycle distribution, and unable to cause short term growth inhibition. However, our p16-ACT expression construct expressed significantly less detectable protein than our wild-type p16 construct and therefore we cannot rule out that we might have seen p16-related function at higher p16-ACT expression levels. Still, this lower p16-ACT protein expression compared to wild-type p16 may be indicative of the relative stability of the two proteins, in which case the major setback of the CC to T mutation on p16-ACT is decreased protein stability rather than decreased function. Notably, p16-ACT did have a limited ability to inhibit colony formation of U2OS cells. This was likely due to the greater incubation times of the colony formation assay (four weeks) compared to the 48 hour short-term growth assays, giving more time for minute differences in growth to develop. As p16-ACT did not bind CDK4, we postulate that p16-ACT is likely acting through a pathway alternative to CDK4 inhibition to slow colony formation, either through its intact N-terminal p16 region or its C-terminal non-p16/non-p14ARF region. Others have reported amino acids 1-80 of p16 alone do not inhibit colony formation [29], so we gather that the p16 region of p16-ACT likely has no legitimate growth-related function on its own. An explanation for the observed growth inhibition may lie in two small regions of sequence homology in the non-p16/non-p14ARF portion of p16-ACT. One of these regions holds homology to a variety of proteins including SIAH1, an E3 ubiquitin protein ligase which shares 60% homology with p16-ACT over an area of 14 amino acids, and CTC1, a protein essential in telomere maintenance which contains 4 areas ranging 14-46 amino acids which hold 40-50% homology to p16-ACT. The second region of homology holds about 80% homology across 6 amino acids to αxβ2, an integrin involved in cell adhesion and complement recognition [30]. It is possible that p16-ACT may be functioning as one of these homologous proteins to cause the inhibition of colony formation we observed.

Considering that *INK4A* frameshift mutations have described across cancer, the function of chimeric p14ARF and p16 proteins is undoubtedly clinically relevant. Cancer-associated *INK4A* mutations often affect either *p14ARF, p16*, or both, leaving the debate open as to whether p14ARF or p16 is more vital in tumor suppression. As the two proteins both modulate similar cellular processes and share a significant amount of genetic sequence, they likely represent complimentary mechanisms in cell cycle regulation and tumor suppression which have evolved to be maintained within a single locus; should one mechanism fail, the other might still be intact to suppress tumor growth. On the other hand, deletion of the entire 9p21 locus, including *p14ARF*, *p16*, as well as the CDK inhibitor *p15*, may be a way for cancer cells to bypass cell cycle regulation with a single deletion. This would explain the high rate of 9p21 deletion in cancers such as T-cell acute lymphoblastic leukemia [31]. However, as the N-terminal p14ARF region of chARF was sufficient to maintain wild-type p14ARF function while p16 required both its N and C terminus to bind CDK4 and inhibit growth, we conclude p14ARF to be the more robust of the two proteins in terms of resistance to mutational inactivation.
